# Clinical characteristics of patients with 2019 coronavirus disease in a non-Wuhan area of Hubei Province, China: a retrospective study

**DOI:** 10.1186/s12879-020-05010-w

**Published:** 2020-04-29

**Authors:** Xin-Ying Zhao, Xuan-Xuan Xu, Hai-Sen Yin, Qin-Ming Hu, Tao Xiong, Yuan-Yan Tang, Ai-Ying Yang, Bao-Ping Yu, Zhi-Ping Huang

**Affiliations:** 1 Department of Hematology, Jingzhou Central Hospital, The Second Clinical Medical College, Yangtze University, No. 60, Jingzhong Road, Jingzhou Central Hospital, Jingzhou, 434020 Hubei Province China; 2 Department of Gastroenterology, Renmin Hospital of Wuhan University, Wuhan, 430060 Hubei Province People’s Republic of China; 3 Key Laboratory of Hubei Province for Digestive System Diseases, Wuhan, China; 4 Department of Infectious Disease, Jingzhou Central Hospital, The Second Clinical Medical College, Yangtze University, Jingzhou, China

**Keywords:** COVID-19, Disease characteristics, Mortality, Respiratory failure, Non-respiratory injury

## Abstract

**Background:**

Since December 2019, the 2019 coronavirus disease (COVID-19) has expanded to cause a worldwide outbreak that more than 600,000 people infected and tens of thousands died. To date, the clinical characteristics of COVID-19 patients in the non-Wuhan areas of Hubei Province in China have not been described.

**Methods:**

We retrospectively analyzed the clinical characteristics and treatment progress of 91 patients diagnosed with COVID-19 in Jingzhou Central Hospital.

**Results:**

Of the 91 patients diagnosed with COVID-19, 30 cases (33.0%) were severe and two patients (2.2%) died. The severe disease group tended to be older (50.5 vs. 42.0 years; *p* = 0.049) and have more chronic disease (40% vs. 14.8%; *p* = 0.009) relative to mild disease group. Only 73.6% of the patients were quantitative polymerase chain reaction (qPCR)-positive on their first tests, while typical chest computed tomography images were obtained for each patient. The most common complaints were cough (*n* = 75; 82.4%), fever (*n* = 59; 64.8%), fatigue (*n* = 35; 38.5%), and diarrhea (*n* = 14; 15.4%). Non-respiratory injury was identified by elevated levels of aspartate aminotransferase (*n* = 18; 19.8%), creatinine (*n* = 5; 5.5%), and creatine kinase (*n* = 14; 15.4%) in laboratory tests. Twenty-eight cases (30.8%) suffered non-respiratory injury, including 50% of the critically ill patients and 21.3% of the mild patients.

**Conclusions:**

Overall, the mortality rate of patients in Jingzhou was lower than that of Wuhan. Importantly, we found liver, kidney, digestive tract, and heart injuries in COVID-19 cases besides respiratory problems. Combining chest computed tomography images with the qPCR analysis of throat swab samples can improve the accuracy of COVID-19 diagnosis.

## Background

Since December 2019, several cases of severe acute respiratory syndrome coronavirus 2 (SARS-CoV-2) infection were first reported the virus has caused an outbreak in a short time by human-to-human transmission throughout China, especially in Hubei Province. The severe contagiousness and rapid disease progression of the 2019 coronavirus disease (COVID-19) have drawn significant global public health attention. As of March 30, 2020, more than 600,000 confirmed cases were reported worldwide, of which Hubei Province is the most affected area with greater than 80,000 cases and thousands of deaths confirmed from COVID-19.

Like severe acute respiratory syndrome (SARS) [1] and Middle East respiratory syndrome (MERS) [2], COVID-19 not only causes infections in the respiratory tract but also in the digestive tract, liver, and heart [3–5]. A considerable proportion of COVID-19 patients develop severe pneumonia, pulmonary edema, acute respiratory distress syndrome, and even multiple organ failure within a short time. The mortality rate of patients in Wuhan was as high as 4.3% at the time of writing this report [5], but may be slightly lower in other areas. The clinical characteristics of COVID-19 patients in non-Wuhan areas of Hubei Province have not previously been described. In this study, we conducted a comprehensive exploration of the epidemiology and clinical features of 91 patients with confirmed COVID-19 admitted to Jingzhou Central Hospital in Jingzhou, one of the most severely affected cities in Hubei Province.

## Methods

### Patients

We retrospectively analyzed patients diagnosed with COVID-19 hospitalized from January 16, 2020 to February 10, 2020. Patients suspected of having COVID-19 were admitted and quarantined, and throat swab samples were collected and tested for severe acute respiratory syndrome coronavirus 2 (SARS-CoV-2) by quantitative polymerase chain reaction assay (qPCR). Patients diagnosed with COVID-19 were enrolled in this study and asked to sign a written informed consent form during hospitalization. This study was approved by the ethics committee of Jingzhou Central Hospital. The patients have not been reported in any other submission by anyone else. The final date of follow-up was February 10, 2020.

### Data collection

Clinical data [age, previous chronic disease, epidemiological history, symptoms, vital signs, computed tomography (CT) images, virus load, laboratory tests, complications, and treatment process] of the 91 patients involved in this study were collected. Acute respiratory distress syndrome was defined according to the Berlin definition [6]. Liver injury was judged by alanine aminotransferase (ALT) and aspartate aminotransferase (AST) levels. Acute kidney injury was identified according to elevated creatinine (Cr) and uric acid levels. The presence of cardiac injury was confirmed if the serum levels of cardiac biomarkers [cardiac troponin i (CTnI), creatine kinase (CK), creatine kinase isoenzyme (CK-MB)] were elevated. The diagnosis of COVID-19 was made by the comprehensive evaluation of epidemiological exposure, symptoms, laboratory tests, chest CT scan, and qPCR analysis.

### QPCR assay for SARS-CoV-2

RNA for further tests was extracted from the throat swab samples, which were collected in virus preservation solution. Next, 5 μl of RNA was added in a PCR reaction tube with 12 μl of nucleic acid amplification reaction solution, 4 μl of enzyme mixture, and 12 μl of ORF1ab/N reaction solution (BioGerm, Shanghai, China). The cycle parameters for PCR amplification assay were set as follows: reverse transcription at 50 °C for 10 min; predenaturation at 95 °C for 5 min; 40 cycles of denaturation at 95 °C for 10 s; and annealing, extending, and collection of fluorescence at 55 °C for 40 s. The open reading frame 1ab (ORF1ab) and nucleocapsid protein (N) gene regions of SARS-CoV-2 were simultaneously tested. Primers for ORF1ab were as follows: forward primer CCCTGTGGGTTTTACACTTAA, reverse primer ACGATTGTGCATCAGCTGA, and the probe 5′-VIC-CCGTCTGCGGTATGTGGAAAGGTTATGG-BHQ1–3′. Primers for N were as follows: forward primer GGGGAACTTCTCCTGCTAGAAT, reverse primer CAGACATTTTGCTCTCAAGCTG, and the probe 5′-FAM- TTGCTGCTGCTTGACAGATT-TAMRA-3′. A cycle threshold value (Ct value) of less than 37 suggested a positive result, while a Ct value of higher than 40 indicated a negative result. And a Ct value between 37 and 40 required retesting.

### Statistical analysis

The Mann–Whitney U test was used to compare continuous variables, while the chi-square test was adopted to compare categorical variables. The statistics were prepared using Excel (Microsoft Corp., Redmond, WA, USA) and GraphPad Prism 5 software (GraphPad Software, La Jolla, CA, USA), and analyzed using SPSS (IBM Corp., Armonk, NY, USA). A *p*-value of less than 0.05 was considered to be statistically significant.

## Results

### Clinical characteristics of COVID-19 patients

We collected clinical information of 91 patients diagnosed with COVID-19 in Jingzhou Central Hospital. Of these patients, 30 (33.0%) were assessed as being severely ill (Table 1). The median age of our study population was 46.0 years, with severe patients being generally older with a median age of 50.5 years relative to that of 42.0 years among the mild cases (*p* = 0.049). Slightly more than half (53.8%) of the patients were male and there were no significant differences in the sex ratio between the severe and mild cases (*p* = 0.230). Twenty-one patients (23.1%) had one or more coexisting medical conditions, including hypertension, diabetes, chronic obstructive pulmonary disease, kidney disease, and malignancies. Coexisting medical conditions were more prominent in the severe disease group (40% had chronic disease vs. 14.8% in mild disease group; *p* = 0.009). The median duration from onset of symptoms to hospital admission was 4 days. All patients were confirmed as qPCR-positive eventually, while only 73.6% of patients were qPCR-positive when they first received the test. The qPCR-positive rate at different times was not different in the severe and mild disease groups (*p* = 0.884). In addition, the vital signs (pulse rate, temperature, and mean arterial pressure) between the two groups showed no significant differences. Compared to the severe disease group (6.7%), the mild disease group (19.7%) had a higher proportion of discharged cases. Of the two deceased patients who were critically ill, one had lung cancer and the other had hypertension. Both suffered respiratory and acute renal failure on the day of admission. The mortality rate was 2.2%. Taken together, our results supported that older patients with chronic disease were more likely to become critically ill and negative qPCR results could not confidently exclude infection with the virus at symptom onset.

**Table 1:** Baseline characteristics, clinical outcomes of 91 patients hospitalized in Jingzhou Central Hospital with COVID-19

	Total (*n* = 91)	Severe (30, 33.0%)	Mild (61, 67.0%)	*P* value
**Median Age**	46.00	50.50	42.00	0.049
Age<60	75/91 (82.4%)	22 /30 (73.3%)	53/61 (86.9%)	
Age ≥ 60	16 /91 (17.6%)	8/30 (26.7%)	8/61 (13.1%)	
**Sex**				0.230
Male	49/91 (53.8%)	14 /30 (46.7%)	35/61 (57.4%)	
Female	42 /91 (46.2%)	16 /30 (53.3%)	26/61 (42.6%)	
**Chronic disease**	21/91 (23.1%)	12/30 (40.0%)	9/61 (14.8%)	0.009
Hypotension	18	9	9	
COPD	1	1	0	
Diabetes	3	1	2	
Autoimmune disease	1	1	0	
Kidney disease	1	1	0	
Malignancy	3	2	1	
**Median days to be admission**	4	5	4	0.714
**QPCR positive**				0.884
For the first time	67/91 (73.6%)	23/30 (76.7%)	44/61 (72.1%)	
For the second time	11 /91 (12.1%)	3/30 (10.0%)	8/61 (13.1%)	
For the third time	13/91 (14.3%)	4/30 (13.3%)	9/61 (14.8%)	
**Vital signs**				
Median pulse rate	80.00	80.00	82.00	0.677
Median mean arterial pressure	93.30	92.30	37.10	0.944
Median temperature	37.20	37.25	93.30	0.894
**Outcomes**				0.041
Remained in hospital	75/91 (82.4%)	26/30 (86.7%)	49/61 (80.3%)	
Discharged	14/91 (15.4%)	2/30 (6.7%)	12/61 (19.7%)	
Died	2/91 (2.2%)	2/30 (6.7%)	0 (0%)	

COPD chronic obstructive pulmonary disease

**Fig. 1:**
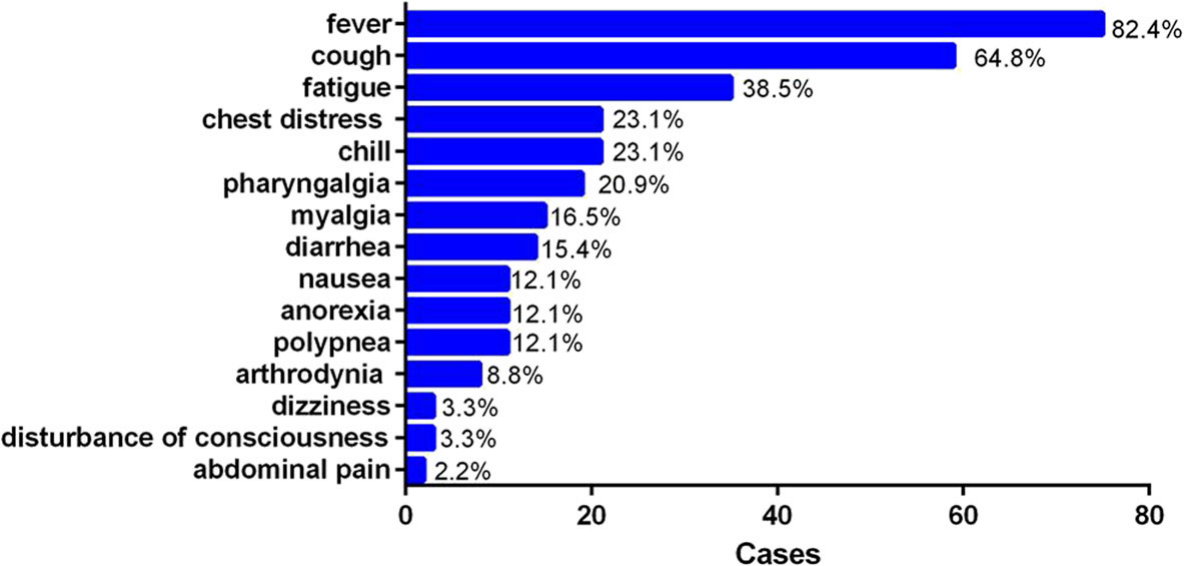
Symptoms of patients with COVID-19 on admission

At data cut off, considering 91 evaluable patients, the most common symptoms were fever (*n* = 75; 82.4%) and cough (*n* = 59; 64.8%), followed by fatigue (*n* = 35; 38.5%), chest distress (*n* = 21; 23.1%), chill (*n* = 21; 23.1%), pharyngalgia (*n* = 19; 20.9%), and myalgia (*n* = 15; 16.5%) (Fig. 1). Additionally, some patients reported gastrointestinal problems, including diarrhea (*n* = 14; 15.4%) and nausea (*n* = 19; 12.1%) (Fig. 1). Symptoms of polypnea and the disturbance of consciousness, as signs of greater disease severity, were reported by 12.1% (*n* = 19) and 3.3% (*n* = 3) of patients, respectively (Fig. 1). Anorexia, arthrodynia, dizziness, and abdominal pain were also found (Fig. 1).

### Baseline characteristics of laboratory tests in COVID-19 patients

To explore the characteristics of laboratory tests from our patients with COVID-19, the baseline hematological and biochemical indices of the 91 patients were analyzed (Table 2). On admission, 51.6% of patients had lymphopenia, which was more prominent in mild cases (Table 2). Elevated CK levels were observed in 15.4% of patients, while elevated ALT and AST levels were recorded in 11.0 and 19.8% of patients. Severe cases had more prominently elevated Cr (16.7% vs. 0%; *p* < 0.001) and CK (26.7% vs. 9.8%; *p* = 0.018) levels. The prothrombin time was prolonged in 20.9% of patients. Levels of interleukin-6 and C-reactive protein, two biomarkers of inflammation, were increased in 19.8 and 40.7% of patients (Table 2). These laboratory findings indicated that COVID-19 resulted in liver, kidney, and cardiovascular injury.

**Table 2:** Baseline laboratory characteristics of patients with COVID-19

Variables	Total (*n* = 91)	Severe (30, 33.0%)	Mild (61, 67.0%)	*P* value
WBC (×10^9/L)				0.127
<4	27/91 (29.7%)	7/30 (23.3%)	20/61 (32.8%)	
4–10	53/91 (58.2%)	18/30 (60.0%)	35/61 (57.4%)	
>10	11/91 (12.1%)	5/30 (16.7%)	6/61 (9.8%)	
Neutrophil (×10^9/L)				0.467
≤ 6.3	80/91 (87.9%)	26/30 (86.7%)	54/61 (88.5%)	
>6.3	11/91 (12.1%)	4/30 (13.3%)	7/61 (11.5%)	
Monocytes (×10^9/L)				0.190
≤ 0.6	66/91 (72.5%)	20/30 (66.7%)	46/61 (75.4%)	
>0.6	25/91 (27.5%)	10/30 (33.3%)	15/61 (24.6%)	
Lymphocytes (×10^9/L)				0.022
≤ 1.1	47/91 (51.6%)	11/30 (36.7%)	36/61 (59.0%)	
>1.1	44/91 (48.4%)	19/30 (63.3%)	25/61 (41.0%)	
CTnI (μg/L)				0.277
≤ 0.01	85/88 (96.6%)	28/30 (93.3%)	57/58 (98.3%)	
>0.01	3/88 (3.4%)	2/30 (6.7%)	1/58 (1.7%)	
CK (U/L)				0.018
≤ 190	77/91 (84.6%)	22/30 (73.3%)	55/61 (90.0%)	
>190	14/91 (15.4%)	8/30 (26.7%)	6/61 (9.8%)	
CK-MB (U/L)				0.099
≤ 24	87/91 (95.6%)	27/30 (90.0%)	60/61 (98.4%)	
>24	4/91 (4.4%)	3/30 (10.0%)	1/61 (1.6%)	
ALT (U/L)				0.112
≤ 50	81/91 (89.0%)	25/30 (83.3%)	56/61 (91.8%)	
>50	10/91 (11.0%)	5/30 (16.7%)	5/61 (8.2%)	
AST (U/L)				0.124
≤ 40	73/91 (80.0%)	22/30 (73.3%)	51/61 (83.6%)	
>40	18/91 (19.8%)	8/30 (26.7%)	10/61 (16.4%)	
Cr (μmol/L)				0.000
≤ 97	86/91 (94.5%)	25/30 (83.3%)	61/61 (100%)	
>97	5/91 (5.5%)	5/30 (16.7%)	0/61 (0%)	
UA (μmol/L)				0.202
≤ 417	86/91 (94.5%)	27/30 (90.0%)	59/61 (96.7%)	
>417	5/91 (5.5%)	3/30 (10.0%)	2/61 (3.3%)	
PT (s)				0.100
≤ 12.8	72/91 (79.1%)	22/30 (73.3%)	50/61 (82%)	
>12.8	19/91 (20.9%)	8/30 (26.7%)	11/61 (18.0%)	
APTT(s)				0.202
≤ 36.5 s	86/91 (94.5%)	27/30 (90.0%)	59/61 (96.7%)	
>36.5	5/91 (5.5%)	3/30 (10.0%)	2/61 (3.3%)	
D-Dimer (ng/ml)				0.484
≤ 232	78/82 (95.1%)	28/30 (93.3%)	50/52 (96.2%)	
>232	4/82 (4.9%)	2/30 (6.7%)	2/52 (3.8%)	
IL-6 (ng/ml)				0.485
≤ 7	73/91 (80.2%)	24/30 (80.0%)	49/61 (80.3%)	
>7	18/91 (19.8%)	6/30 (20.0%)	12/61 (19.7%)	
CRP (mg/ml)				0.240
≤ 10	56/91 (61.5%)	20/30 (66.7%)	36/61 (59.0%)	
>10	35/91 (38.5%)	10/30 (33.3%)	25/61 (41.0%)	
PCT (ng/ml)				0.106
≤ 0.5	85/89 (95.5%)	27/30 (90.0%)	58/59 (98.3%)	
>0.5	4/89 (4.5%)	3/30 (10.0%)	1/59 (1.7%)	

Data are n/N (%), where N is the total number of patients with available data. *p* values comparing severe and mild groups used Chi-square test, Chi-square with Yates’ correction, or Fisher’s exact test

*WBC* White blood cell, *CTnI* Cardiac troponin I, *CK* Creatine kinase, *CK-MB* Creatine kinase isoenzyme, *ALT* Alanine aminotransferase, *AST* Aspartate aminotransferase, *Cr* Creatinine, *UA* Uric acid, *PT* Prothrombin time, *APTT* Activated partial thromboplastin time, *IL-6* Interleukin 6, *CRP* C-reactive protein, *PCT* Procalcitonin

### Non-respiratory system injury with COVID-19

Interestingly, separate from damage to the respiratory system, COVID-19 patients showed signs of multiple organ injury on admission, including 18 cases (19.8%) of liver injury; 14 cases (15.4%) of cardiovascular damage with abnormal increases in troponin, CK, or CK-MB levels; five cases (5.5%) of acute renal injury; and 19 cases (20.9%) of poor coagulation function (Table 3). Together, 28 patients (30.8%) suffered non-respiratory system injury, with an especially higher rate (50% vs. 21.3%; *p* = 0.003) in the severe disease group (Table 3). Further analysis revealed that severe patients tended to suffer damage to the cardiovascular system (26.7% vs. 9.8%; *p* = 0.04) and renal function (16.7% vs. 0%; *p* = 0.003) (Table 3). Angiotensin-converting enzyme II (ACE2) was proved to be the cell receptor of COVID-19 [7], the same as SARS infection [8]. We performed bioinformatics analysis on the expression of ACE2 receptors in different normal tissues sourced from the cancer microarray database Oncomine, as shown in Additional file 1. The data indicated that the highest level of ACE2 was present in the ileum, followed by in the testis, diaphragm, heart, kidneys, seminal vesicle, colon, and respiratory tract. Hence, we speculated that the high ACE2 expression levels in the ileum, heart, kidneys, and colon caused the virus to pursue direct infection in these specific organs, which might explain the high rate of multiple organ damage caused by COVID-19.

**Table 3:** Non-respiratory system injury with COVID-19

	Total (*n* = 91)	Severe (30, 33.0%)	Mild (61, 67.0%)	*P* value
Total	28/91 (30.8%)	15/30 (50.0%)	13/61 (21.3%)	0.003
Cardiovascular	14/91 (15.4%)	8/30 (26.7%)	6/61 (9.8%)	0.040
Digestive tract	14 /91 (15.4%)	6 /30 (20.0%)	8 /61 (13.1%)	0.287
Liver	18 /91 (19.8%)	8/30 (26.7%)	10/61 (16.4%)	0.189
Renal	5/91 (5.5%)	5/30 (16.7%)	0 (0)	0.003
Coagulation function	19/91 (20.9%)	8/30 (26.7%)	11/61 (18.0%)	0.246

### Typical CT images

**Fig. 2:**
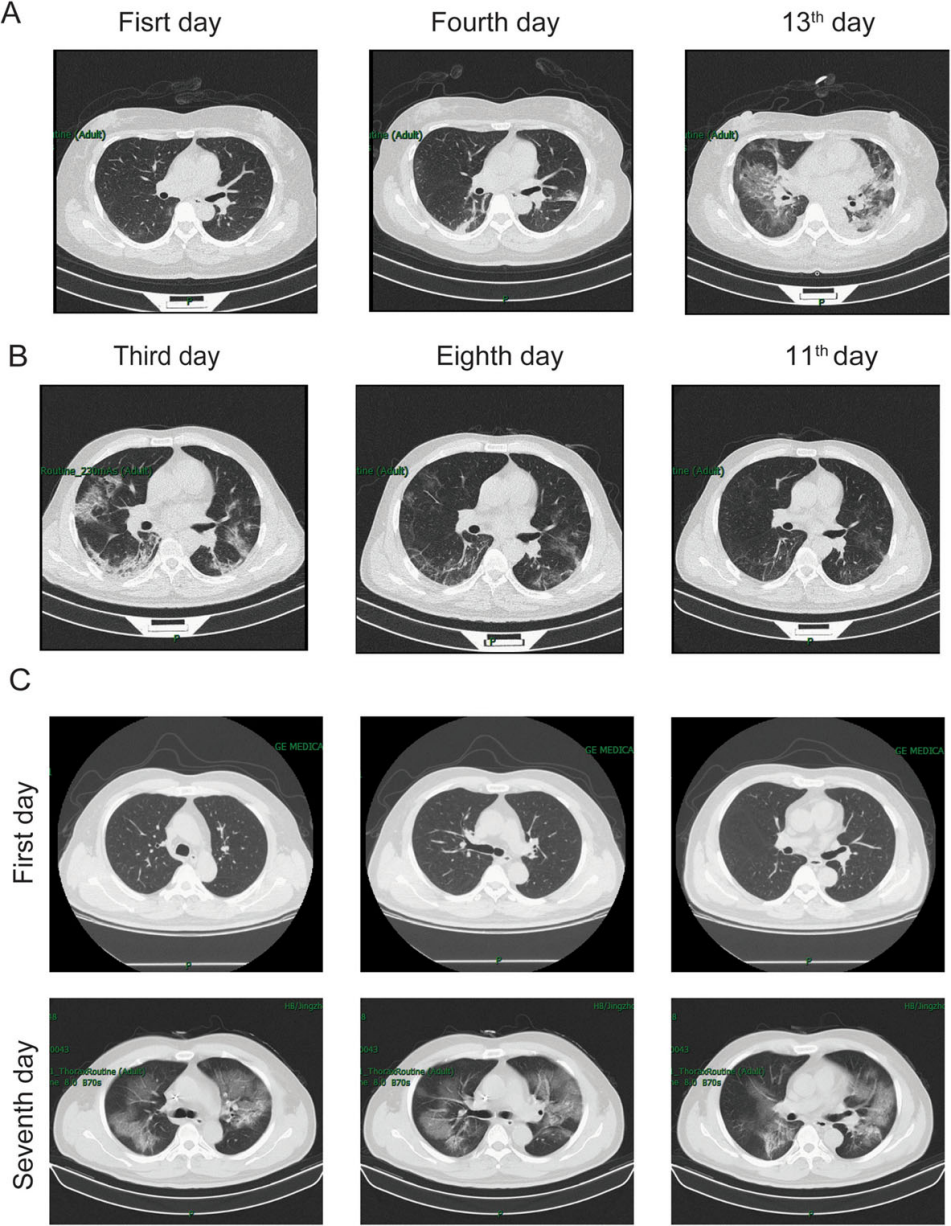
Chest CT images of patients with COVID-19. **a** Chest CT images of case 1 on different days after admission. **b** Chest CT images of case 2 on different days after admission. **c** Chest images on different days of a patient who died of respiratory failure

All of the patients in our study presented exudative changes, with different degrees of patchy consolidation or ground-glass opacities, on CT images (Fig. 2a–c). The processing of CT images (Fig. 2a) revealed that the chest presented only scattered dotted shadows on the first day of admission, focal exudation on the fourth day, and diffuse ground-glass shadows on the 13th day. A good example of a case gradually improved with effective therapy is presented in Fig. 2b. In addition, there were patients whose condition progressed rapidly within 1 week (Fig. 2c). On the first day of admission, the chest CT scan was basically normal. However, on the seventh day, the chest CT scan showed patchy high-density shadow and diffused ground glass density shadow in both lungs. Soon after, this patient died of multiple organ failure (Fig. 2c). These results suggest that CT imaging is an important method in differential diagnosis and evaluating the severity of COVID-19.

### Main treatment measures of COVID-19 patients during hospitalization

Most patients received antiviral therapy (*n* = 81; 89.0%), glucocorticoid therapy (*n* = 79; 86.8%), and antibacterial therapy (*n* = 90; 98.9%), which was given in all severe cases (*n* = 30; 100.0%) (Table 4). Oseltamivir, lopinavir/ritonavir, and umifenovir were common antiviral drugs used in our treatment protocols. In our study, 26.4% of patients were treated with oseltamivir antiviral therapy (Table 4). Lopinavir/ritonavir was more likely to be used in the mild disease group (53.3% vs. 78.7%; *p* = 0.013), while umifenovir was more likely to be used in the severe disease group (73.3% vs. 50.8%; *p* = 0.033) (Table 4). Supportive treatment measures, including oxygen therapy (*n* = 29; 31.9%), mechanical ventilation (*n* = 5; 5.5%), the infusion of immunoglobulin (*n* = 35; 38.5%), and continuous renal replacement therapy (*n* = 3; 3.3%), were more likely to be applied to patients in the severe disease group (*p* < 0.05) (Table 4).

**Table 4:** Treatment of patients with COVID-19

	Total (*n* = 91)	Severe (30, 33.0%)	Mild (61, 67.0%)	*P* value
**glucocorticoid**	79/91 (86.8%)	25/30 (83.3%)	54/61 (88.5%)	0.399
**antiviral therapy**	81/91 (89.0%)	24/30 (80.0%)	57/61 (93.4%)	0.061
oseltamivir	24/91 (26.4%)	6/30 (20.0%)	18/61 (29.5%)	0.240
lopinavir/ritonavir	64/91 (60.3%)	16/30 (53.3%)	48/61 (78.7%)	0.013
umifenovir	53/91 (58.2%)	22/30 (73.3%)	31/61 (50.8%)	0.033
**antibacterial therapy**	90/91 (98.9%)	30/30 (100.0%)	60/61 (98.4%)	0.670
cephalosporin	27/91 (29.7%)	12/30 (40.0%)	15/61 (24.6%)	0.103
fluoroquinolones	84/91 (92.3%)	27/30 (90.0%)	57/61 (93.4%)	0.421
carbapenems	2/91 (2.2%)	2/30 (6.7%)	0 (0)	0.059
**immunoglobulin**	35/91 (38.5%)	16/30 (53.3%)	19/61 (31.2%)	0.035
**oxygen therapy**	29/91 (31.9%)	17/30 (56.7%)	12/61 (19.7%)	0.001
**mechanical ventilation**	5/91 (5.5%)	5/30 (16.7%)	0 (0)	0.003
**CRRT**	3/91 (3.3%)	3/30 (10.0%)	0 (0)	0.033

*CRRT* Continuous renal replacement therapy

## Discussion

According to the data reported, the mortality rate in Wuhan (4.3%) [5] is indeed higher than in other areas. As Jingzhou ranks among the top three cities that have the most immigrant population from Wuhan but does not confront the same challenges in Wuhan, we contend that the cases described in this paper are more representative of the course of COVID-19. There are two main reasons accounting for the higher mortality rate reported in Wuhan. Although all COVID-19 patients are treated in public hospitals and all expenses are borne by the government, patients in Wuhan could not obtain prompt and adequate treatment as a result of the area hospitals being overloaded with large numbers of patients in a short time. Further, we found that patients in the Jingzhou Central Hospital were often younger, with a median age of 46.0 years relative to that of 56.0 years in Wuhan. Also, there were fewer patients with coexisting chronic diseases in this study, which assisted in lowering the mortality rate [5].

Not all of our patients were qPCR-positive after throat swab sampling during their first test. It took three times to obtain a positive qPCR result for 14.3% of the patients in our study. False negatives often exist during qPCR testing. All patients presented typical CT imaging changes during the study, thus we could establish a clinical diagnosis decision using CT before positive qPCR results were obtained. Hence, CT imaging is a favorable means for diagnosing COVID-19 as well as evaluating the severity of the disease. In sum, the confirmation of COVID-19 should be dependent upon the comprehensive analysis of epidemiological exposure, symptoms, laboratory tests, qPCR, and CT imaging.

Based on the symptoms and laboratory examinations of our patients, we found that, in addition to the respiratory tract, the digestive tract, liver, renal function, and cardiovascular system were affected. The mechanism of multiple organ damage in the context of COVID-19 infection is currently unclear. The virus enters into the host cells by the recognition of spike glycoproteins. Accumulated evidence has shown that ACE2 is the cell receptor of choice for SARS-CoV-2, same as in the SARS-CoV infection, which means that the virus infects cells expressing ACE2 [8–11]. It was also reported that anti-ACE2 therapy blocked coronavirus replication during in vitro experiments [11]. It is even proposed that angiotensin receptor 1 blockers might be a treatment option for SARS [12, 13], but there remains a lack of practice basis in this regard at present. ACE2 was initially thought to be expressed only in the heart, kidneys, and testis, but has now been found to be widely expressed in the lungs, brain, and digestive tract [8–10]. These results, together with the bioinformatics analysis in our study, might explain why the COVID-19 caused multiple organ damage. Other possible reasons, including hypoxia caused by respiratory failure and the immune response caused by virus, might also account for the multiple organ damage.

Due to the lack of effective antiviral drugs, some patients got worse and developed respiratory failure in seven to 10 days. Almost all the patients in this study received antibacterial agents, 89.01% received antiviral therapy and 86.81% received glucocorticoid therapy. Oseltamivir is used to treat the influenza virus by inhibiting neuraminidase. The use rate of oseltamivir varies across different studies from 35.8% in the study of Zhong et al. [14] to 89.9% in that by Wang et al. [5]. In our study, 26.4% of patients were treated with oseltamivir. At the beginning of the disease course, it can be difficult to distinguish the symptoms of patients with COVID-19 from those with influenza. Further, some patients tested positive for influenza virus antibodies, so oseltamivir antiviral therapy was used. In most cases, this drug was used in combination with other antiviral (lopinavir/ritonavir and umifenovir) and antibacterial agents. So far, there are no specific antiviral agents available to treat SARS-CoV-2, SARS, or MERS. More prospective studies on specific antiviral therapy might help overcome this challenge.

Although the use of glucocorticoids in virus pneumonia is still very controversial, these medications are widely used in clinical practice. The 86.81% frequency rate for use in our study is higher than in other reports [3–5]. It is yet to be confirmed that the lower mortality in our study is correlated with the higher glucocorticoid utility ratio. Chen et al. retrospectively analyzed 401 patients, including 249 critically ill patients, showing that glucocorticoids were effective in controlling the inflammatory response caused by SARS [15]. However, the multivariate analysis of another retrospective analysis suggested that corticosteroid therapy was significantly associated with a 20.7 times higher intensive care unit occupancy rate among patients with SARS relative to SARS patients who did not receive corticosteroids [16]. Thus, further prospective investigations are required to explore the benefits and side effects of glucocorticoid treatments in patients with viral pneumonia.

## Conclusion

Our single-center study of 91 cases of confirmed COVID-19 sampled from a distinct but representative location (Jingzhou) other than Wuhan feeds into the ongoing efforts to understand COVID-19, which should benefit the diagnosis, therapy, and control of the spread of the disease. Further prospective investigations of effective antiviral therapies and SARS-CoV-2 vaccines may help remove this challenge.

## Supplementary information

**Additional file 1 Figure S1..** ACE2 distribution in different normal tissues from Oncomine (Nucleotide Acc No.:AA416585).

## Data Availability

The datasets generated and/or analyzed during the current study are not publicly available due individual privacy of patients could be compromised, but are available from the corresponding author on reasonable request.

## Abbreviations

COVID-19: 2019 coronavirus disease
SARS: Severe acute respiratory syndrome
MERS: Middle East respiratory syndrome
SARS-CoV-2: Severe acute respiratory syndrome coronavirus 2
qPCR: Quantitative polymerase chain reaction
CT: Computed tomographic
WBC: White blood cell
CTnI: Troponin i
CK: Creatine kinase
CK-MB: Creatine kinase isoenzyme
ALT: Alanine aminotransferase
AST: Aspartate aminotransferase
Cr: Creatinine
UA: Uric acid
PT: Prothrombin time
APTT: Activated partial thromboplastin time
IL-6: Interleukin 6
CRP: C-reactive protein
PCT: Procalcitonin
ACE2: Angiotensin converting enzyme II
CRRT: Continuous renal replacement therapy
