# Supplementary material for: Clinical characteristics of patients with 2019 coronavirus disease in a non-Wuhan area of Hubei Province, China: a retrospective study
Source: BMC Infect Dis. 2020 Apr 29;20:311. doi: 10.1186/s12879-020-05010-w (PMC7188494; doi:10.1186/s12879-020-05010-w)
Supplement: Supplementary file 1 — Additional file 1 Figure S1.. ACE2 distribution in different normal tissues from Oncomine (Nucleotide Acc No.:AA416585). [file 12879_2020_5010_MOESM1_ESM.pdf]

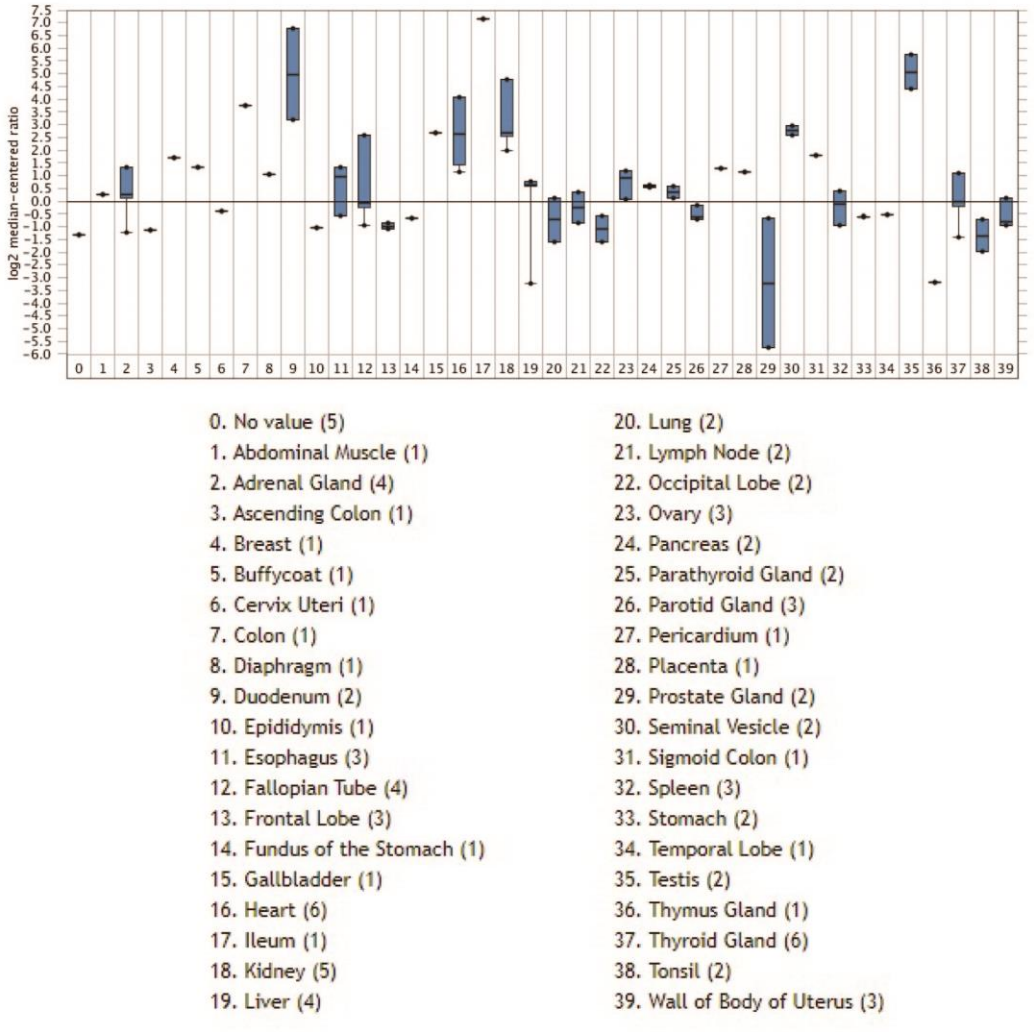

Supplementary Fig 1. ACE2 distribution in different normal tissues from Oncomine (Nucleotide Acc No.:AA416585).
